# Pesticide toxicogenomics across scales: *in vitro* transcriptome predicts mechanisms and outcomes of exposure *in vivo*

**DOI:** 10.1038/srep38131

**Published:** 2016-12-01

**Authors:** Immacolata Porreca, Fulvio D’Angelo, Lucia De Franceschi, Alessandro Mattè, Michele Ceccarelli, Achille Iolascon, Alberto Zamò, Filomena Russo, Maria Ravo, Roberta Tarallo, Marzia Scarfò, Alessandro Weisz, Mario De Felice, Massimo Mallardo, Concetta Ambrosino

**Affiliations:** 1 IRGS, Biogem, Via Camporeale, 83031, Ariano Irpino, Avellino, Italy; 2 Department of Medicine, University of Verona-AOUI Verona, Policlinico GB Rossi, P.Le L. Scuro, 10, 37134 Verona, Italy; 3 Department of Science and Technology, University of Sannio, Via Port’Arsa 11, 82100, Benevento, Italy; 4 Molecular Medicine and Medical Biotechnologies, University of Naples “Federico II Napoli, Italy; 5 Department of Diagnostics and Public Health, University of Verona-AOUI Verona, Policlinico GB Rossi, P.Le L. Scuro, 10, 37134 Verona, Italy; 6 Laboratory of Molecular Medicine and Genomics, Department of Medicine, Surgery and Dentistry ‘Schola Medica Salernitana’, University of Salerno, Baronissi, Salerno, Italy; 7 IEOS-CNR, Via Pansini 6, 80131 Napoli, Italy

## Abstract

*In vitro* Omics analysis (i.e. transcriptome) is suggested to predict *in vivo* toxicity and adverse effects in humans, although the causal link between high-throughput data and effects *in vivo* is not easily established. Indeed, the chemical-organism interaction can involve processes, such as adaptation, not established in cell cultures. Starting from this consideration we investigate the transcriptomic response of immortalized thyrocytes to ethylenthiourea and chlorpyrifos. *In vitro* data revealed specific and common genes/mechanisms of toxicity, controlling the proliferation/survival of the thyrocytes and unrelated hematopoietic cell lineages. These results were phenotypically confirmed *in vivo* by the reduction of circulating T4 hormone and the development of pancytopenia after long exposure. Our data imply that *in vitro* toxicogenomics is a powerful tool in predicting adverse effects *in vivo*, experimentally confirming the vision described as Tox21c (Toxicity Testing in the 21st century) although not fully recapitulating the biocomplexity of a living animal.

Toxicogenomics aims to define the molecular patterns predicting the *in vivo* toxicity, the adverse effects in humans, or the individual susceptibility to chemicals, allowing a better extrapolation of animal data to humans^1^. Since the publication of a report entitled “Toxicity Testing in the 21st Century: A Vision and a Strategy”, several researchers have been aiming to optimise the application of Omics technologies to *in vitro* cell systems, with a differentiated phenotype, for predictive toxicology^2^. Nowadays, there is a strong need for a rapid mechanism-based strategy in risk assessment, achievable in an easier manner in cell lines. This strategy could be used for the decision to opt out or to proceed with further animal tests, matching the need to apply the ‘3Rs’ concept (Replacement, Reduction and Refinement). At present, a practical application of the Tox21c vision is still far away because of the limited confidence in the new method as the causal link between *in vitro* data, obtained with new technologies, and adverse effects, tested *in vivo*, is difficult to establish. The crucial confidence can be reached by simultaneously evaluating phenotypic and molecular endpoints, the latter achievable through *in vitro* Omics analyses^3^,^4^, considering that the chemical-cell/organ interaction *in vivo* can be characterized by compensation and adaptive response not necessarily developed in differentiated cell cultures.

Hypothyroidism is increasing worldwide^5^ and several modifiable factors, i.e. diet and environmental pollutants, have been involved. Thyroid Disrupting Chemicals (THDCs) exert their effects on function and regulation of the thyroid tissue, especially during the early-life stages^6^,^7^.

Thyroid dysfunction has been associated to pesticides exposure in different epidemiological^8^,^9^ and experimental studies^10^,^11^, although a debate is heated. The traditional toxicology approach, based on long-lasting *in vivo* experiments, did not provide exhaustive information about the mechanism of action of THDCs^7^,^12^.

We have recently reported that THDCs toxicity of low-dose bisphenol-A (BPA) can be highlighted investigating directly the expression of thyroid specific genes in rat immortalized thyrocytes^13^ while unpredicted mechanisms of toxicity are evidenced by *in vitro* toxicogenomics^14^.

Here, we practically applied the Tox21c suggestions in the investigation of the dose-dependent effects of ethylenethiourea (ETU) and chlorpyrifos (CPF), both exerting THDCs activity^10^,^11^,^15^, starting from the transcriptomic analyses of exposed immortalized rat thyrocytes. We aimed to: a) consolidate the suggestion that *in vitro* toxicogenomics could draft the *in vivo* cell response; b) identify the thyroid signature and mechanisms of toxicity for *in vivo* validation; c) highlight *in vitro* cell type unrelated outcomes evaluable *in vivo*, as here shown for the haematopoiesis.

## Results

### Transcriptomic analyses in CPF and ETU treated cells

*In vitro* toxicogenomic experiments were performed to generate data to be verified *in vivo.* We assessed the low-dose and the mixture effects of CPF and ETU in PCCl3 cells, rat immortalized thyrocytes, considered a valuable model for studying thyroid cell function and transformation *in vitro*^16^. Our interest in ETU and CPF is due to their ability to impair thyroid physiology^10^,^11^, documented at morphological but not at molecular level. Considering the non-monotonic dose-response curves of the THDCs, we tested the transcriptomic effects of the pesticides at different concentrations.

Experimentally, PCCl3 cells were exposed for one week to different doses of both pesticides as reported in Table 1 and to their combinations. In the following we use the H, M, L abbreviations to state high, medium and low concentrations, respectively. The chosen concentrations are in or below the range of the doses tested in immortalized thyrocytes for ETU^17^ and other cell systems for CPF^18^. Since humans are usually co-exposed, we also treated the cells with the mixtures of ETU and CPF to evaluate possible additive/synergic effects. Of note, the used concentrations are within the range stated for non-exposed population (low and medium concentrations), and for exposed workers (high concentrations) in reports from the Centers for Disease Control and Prevention (CDC) for CPF^19^ and ETU^20^, although lower levels of both compounds have been recently detected in human fluids^21^.

**Figure 1:**
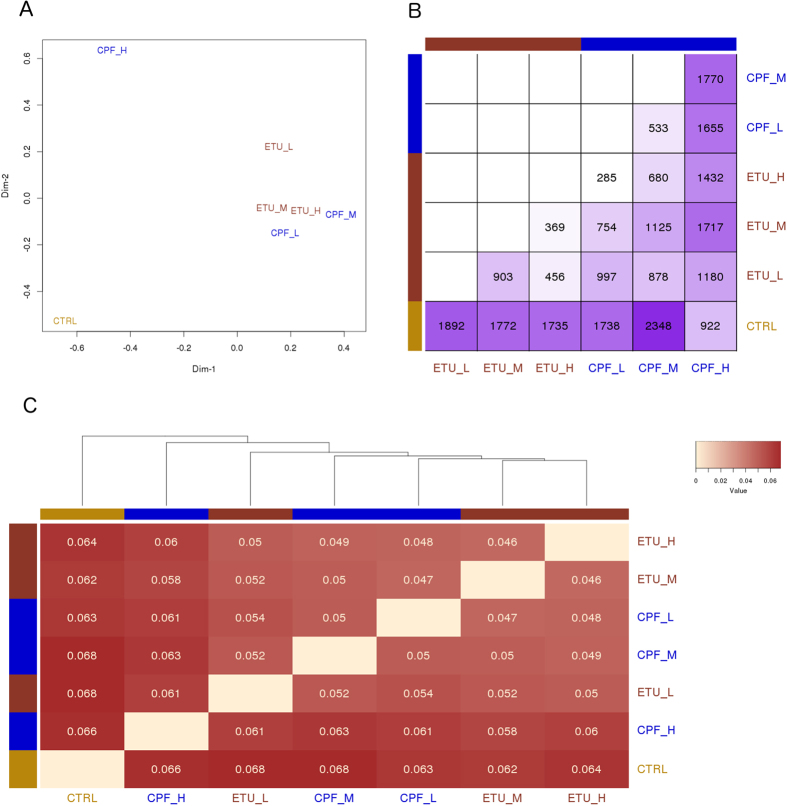
Overview of gene expression analysis. (**A**) Multi-dimensional scaling plot shows the two main components explaining the differences across the conditions. (**B**) Heatmap representing the number of significant differentially expressed genes (corrected *p*-value ≤ 0.05) for each contrast. (**C**) Jensen-Shannon distances between conditions were calculated to verify their similarities across all genes and represented by heatmap. Pairs with fairer colour are more similar and are represented nearer in the dendrogram.

The genome-wide view of the PCCl3 transcriptome was obtained by high-throughput RNA-sequencing (RNAseq) data analysis. The differential gene expression was synthesized in only two relative values for each condition to draw a multi-dimensional scaling plot (Fig. 1A). A deep impact of both compounds on the transcriptome was observed. CPF had the stronger concentration-dependent effect as highlighted also in the related overview matrix (Fig. 1B), showing an increased number of deregulated genes in CPF treated samples vs control. ETU had a smaller concentration-dependent effect. Pairwise similarities between conditions were measured as JS distances across all genes^22^, reported in the heatmap and used to build a dendrogram between the conditions for CPF and ETU treatments (Fig. 1C). The JS distance between CPF-H and CPF-M (0.0625) or CPF-L (0.0609) was similar to the control (0.0662), underlining the non- monotonic concentration-response relationship. The reduced JS distance in co-exposed samples, MxM, LxL, indicated an alleviated impact on the transcriptome, also evidenced in Fig. 1B. Tested genes passing the fold change (FC) and corrected *p-*value filters (absolute log_2_FC ≥ 1, corrected *p-*value ≤ 0.05) were defined as Differentially Expressed Genes (DEGs) and represented in volcano plots (Supplementary Fig. 1).

**Figure 2:**
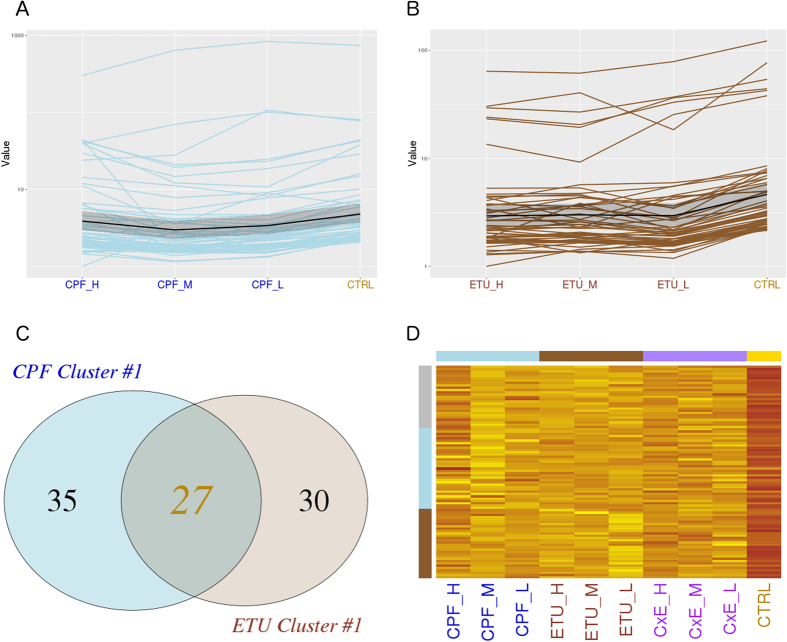
CPF and ETU clusters #1 expression profiling. DEGs were partitioned by k-means clustering analysis. The expression profiles of genes included in the most significant cluster is reported as line plot across the different doses for CPF (**A**), for ETU (**B**) and, finally, compared by Venn diagram (**C**). The expression profiles of selected common genes are represented by heatmap for CPF and ETU, including the different doses and combination treatments (**D**).

Gene cluster analysis was applied on DEGs retrieved at each dose, specifically for ETU and for CPF to study the dose-dependent effects of the two compounds. Partitioning of DEGs into clusters with similar expression profiles was achieved through K-means clustering analysis using JS distance as metric. Three different gene clusters were observed among the CPF-DEGs and three for ETU-DEGs (data not shown). Molecular signatures of toxicity were identified in the most significant cluster (cluster #1), including the higher number of genes. CPF-cluster #1 was composed of 62 transcripts (Fig. 2A and Supplementary Table 1) inhibited in CPF-L and/or CPF-M condition and not regulated at CPF-H. ETU-cluster #1 (Fig. 2B and Supplementary Table 2) consisted of 59 DEG, whose transcripts decreased at ETU-M and increased again at ETU-H. The two clusters were compared to selected common and specific genes through a Venn diagram (Fig. 2C). We defined a main signature of thyroid toxicity represented by 27 common DEGs and specific signatures composed of genes exclusively included in the CPF- or ETU- list. Their expression profiles were reported by heatmap for each dose of pesticides and their mixture (Fig. 2D). The heatmap showed the differences in the expression of the gene signatures and, strikingly, their deep changes in each condition of co-exposure.

**Figure 3:**
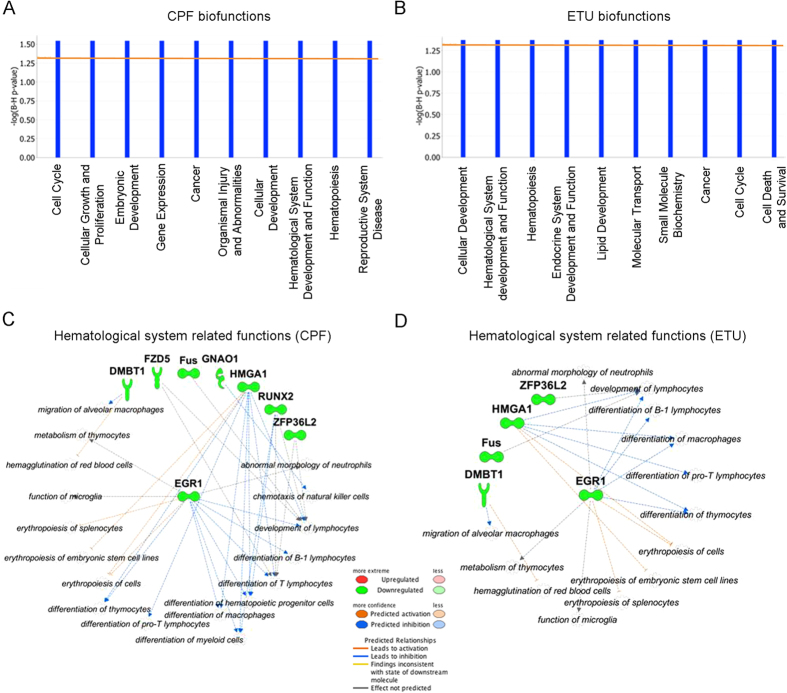
CPF and ETU clusters #1 biofunctions. Bio-functions identified by IPA analysis of CPF- cluster #1 (**A**) and ETU- cluster #1 (**B**). The left y-axis value is a significant score, −log_10_(BH corrected *p-*value), and the orange line evidences the threshold level (corrected p-value ≤ 0.05). Significant hematological system related functions are represented as molecular networks to evidence their relationships with deregulated genes included in CPF-cluster #1 (**C**) and ETU-cluster #1 (**D**).

Insights into mechanisms of toxicity and unpredicted outcomes were obtained by Ingenuity Pathway Analysis (IPA), revealing the biological/toxicological functions over-represented in each gene cluster, in particular CPF- and ETU- cluster #1. The number of non-annotated genes in the rat genome assembly, used for read mapping (RGSC 5.0/rn5), was unfortunately too high to execute a functional analysis. Thus, we compared the sequences of non-annotated rat DEGs with human genes to label them with the corresponding human gene annotation having a high percentage of identity. We performed the IPA analyses on the new lists to identify the enriched biofunctions. The ten most significant biofunctions obtained from the CPF- cluster #1 and ETU- cluster #1 are reported in Fig. 3A and B, respectively. The low *p-*value was not surprising considering the small number of annotated genes. Unexpectedly, no damage of highly specific thyroid cell function was shown, although the impairment of thyroid cell growth was suggested by the biofunction “The Cell Cycle, Endocrine System Development and Function” (G2/M delay, CPF *p-*value = 2,86E-02 and ETU *p-*value = 4,24E-02, and S phase entry CPF *p-*value = 3,84E-02 ETU *p-*value = 4,84E-02). The same IPA analyses identified “Cellular Development, Hematological System Development and Function, Hematopoiesis” as a common deregulated biofunction (*p-*value = 2,86E-02 and *p-*value = 4,24E-02, respectively) for both compounds. The related gene networks are shown in Fig. 3C (CPF) and Fig. 3D (ETU).

Overall, *in vitro* transcriptomics distinguished between CPF- and ETU- treated samples, confirming a non-monotonic concentration-dependent effect (U-inverted shape) and the differences in co-exposure conditions. The signatures of thyroid toxicity common or specific for CPF and ETU were highlighted and both were modified by the co-exposure. The bioinformatics analysis suggested that both compounds could impair the growth of thyrocytes and predicted the hematopoietic dysfunctions. The bioinformatics predictions as well as the identified signatures were further assessed *in vivo*.

### *In vitro* toxicogenomics points to mechanisms of thyreo-toxicity *in vivo*

To validate *in vivo* the *in vitro* toxicogenomics results, the mice were exposed from conception (GD 0) to CPF and ETU (10 mg/kg/day, 1 mg/kg/day and 0.1 mg/kg/day) and their combinations, as detailed in material and methods. Briefly, the dams were exposed to CPF and ETU, by feeding and watering respectively. We continued the exposure by the mother till the weaning and directly lifelong. ETU and CPF doses have been chosen to avoid systemic toxicity up to previously published reports^10^,^11^. For the mixture study we combined the lower dose of CPF with the lower dose of ETU (both 0, 1 mg/kg/day), known to not lead to thyroid phenotypic alterations to verify any additive effect, and the higher dose of CPF with the higher dose of ETU. The route and window of exposure have been selected in order to be relevant for humans. We have conducted a long-term exposure supposing that the damage of the thyroid due to foetal exposure could be compensated and that continued exposure would be necessary to reveal thyroid injuries in adulthood. As thyroid disorders are more common in women^5^, we analysed the thyroid molecular and phenotypic damages in females. General phenotypic analysis of the exposed females did not show any significant difference in survival or body weight (data not shown). Observed birth counts suggested reduced fertility of F1 generation, currently under investigation. Among the common deregulated genes, we assessed the expression of *Egr1*, *Hmga1* and *Zfp36l2* at post natal day (PND) 180 (Supplementary Table 3) and PND 360 (Table 2). The first two were reported as cell cycle regulators in thyroid and other cell types^23^,^24^, while the third was enriched in the thyroid primordium^25^. The exposure to CPF and ETU decreased their expression at PND 360, even if their inhibition was statistically significant only in some treatments (Table 2). The absence of a linear dose-response curve for the chemicals administrated was not surprising as both compounds are considered endocrine disruptors. A trend towards down-regulation was not observed at an earlier time point (Supplementary Table 3). We also tested the expression of CPF-DEGs (*Runx2, Gnao1* and *Fzd5*) and ETU-DEGs (*Ergic1*, *Zfp524* and *Ifit3*) at PND 360, respectively. The trend towards inhibition was confirmed *in vivo* but the compound specificity was hard to define. Indeed, the specific genes were inhibited with both compounds although the statistical significance was reached only in ETU samples for *Ergic1* and *Zfp524*, identified as ETU specific, and *Gnao1* among the CPF-specific genes.

**Figure 4:**
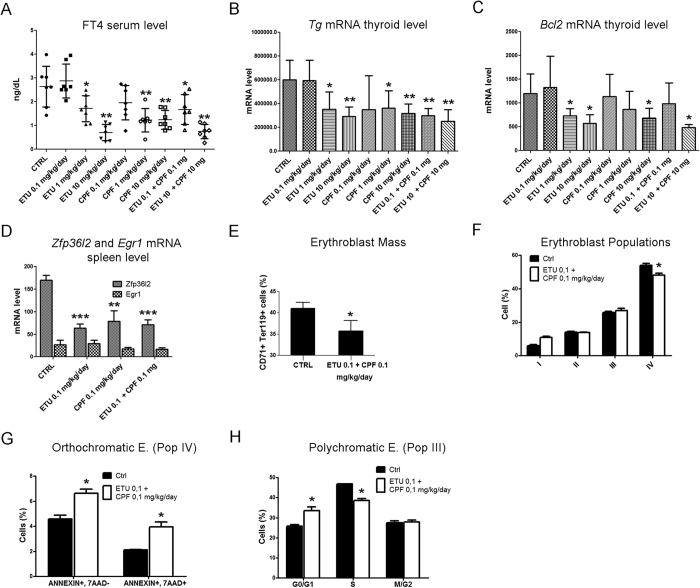
*In vivo* validation of the mechanisms of thyroid toxicity and hematopoietic dysfunction. Thyroid toxicity was verified in females exposed to ETU or CPF (10, 1, 0.1 mg/kg/day) and their combination (10 mg/kg/day, 0.1 mg/kg/day) until PND 360. (**A**) FT4 serum level, each sign is a single mouse. Mean and standard deviation are reported. (**B**) *Tg* and (**C**) *Bcl2* mRNA levels in the thyroid of the same females. (**D**) RT-qPCR analyses of *Zfp36l2* and *Egr1* transcripts in spleen. Data in panels B–D are reported as means ± SD of *Gapdh*-normalized mRNA levels. (**E**) Number of cells CD71 + TER119 + in bone marrow (**F**) Maturation pattern of erythroid precursors by FACS analysis using CD44 and TER119 as surface markers. The pro-erythroblasts (Pop I), basophilic erythroblasts (Pop II), polychromatic erythroblasts (Pop III) and orthochromatic erythroblasts (Pop IV) homogenous populations were gated. (**G**) Amount of Annexin V^+^-7AAD^−^ cells (early apoptosis) and Annexin V^+^-7AAD^+^ (late apoptosis) in sorted Pop IV. (**H**) Cell cycle analysis of Pop III. Data are presented as means ± SD (n = 6). Haematologic dysfunctions reported in panels D-H were verified at CPF and ETU (0.1 mg/kg/day) and their mixture.

The *in vivo* phenotypic validation of thyroid effects was performed determining the free thyroxin (FT4) blood level in treated females. FT4 was reduced at PND 360 (Fig. 4A) but not at PND180 (Supplementary Fig. 2A). Furthermore, thyroglobulin (*Tg*) transcript was inhibited at high and medium doses of both pesticides at PND 360 (Fig. 4B) but not at earlier times (Supplementary Fig. 2B). A synergistic effect was observed when low-dose of both molecules was co-administered. Furthermore, we tested the thyroid expression level of the anti-apoptotic factor *Bcl2* that plays a key role in thyrocyte survival^26^,^27^. *Bcl2* transcript was inhibited by both compounds only at high doses and by their combination at PND 360 (Fig. 4C) but not at PND 180 (Supplementary Fig. 2C).

Overall the results show that the bioinformatics analysis of *in vitro* transcriptome can predict the *in vivo* cell response even if functional predictions are not fully confirmed by *in vivo* thyroid phenotyping. Thus, we have tried to validate the effects on thyrocytes growth/survival *in vitro*. We exposed the PCCl3 to different concentrations of CPF and ETU for 1 week to analyse their proliferation by MTT assay. Subsequently, we blocked their proliferation by thyroid stimulating hormone (TSH) deprivation for 3 days. Then, the cell growth was resumed by hormone addiction. The treatments with CPF and ETU resulted in the reduction in cell number, monitored by MTT, after 72 hrs of stimulation (Supplementary Fig. 3).

The presented data suggest that *in vitro* systems do not fully recapitulate the *in vivo* response because compensation mechanisms are active *in vivo*. Despite that, *in vitro* toxicogenomics predicts mechanism of thyroid toxicity confirmed *in vivo* if long exposures are conducted.

### Toxicogenomics identifies unpredicted effects of the pesticides on hematopoiesis

The inhibition of *Egr1*, *Hmga1* and *Zfp36l2* transcripts was also related to hematological disease by IPA analysis, especially defects in erythropoiesis in CPF (Fig. 3A) and ETU (Fig. 3B).

Hematological dysfunctions have been reported in pesticide applicators^28^ and in treated mice^29^, although a mixture analysis has rarely been conducted. The synergistic effects of CPF and ETU on the hematological compartment were analysed in the sacrificed females exposed to 0.1 mg/kg/day of each compound administrated as single molecule or in combination. The hematologic parameters in the females within each treatment group were investigated and are reported in Table 3. Haemoglobin levels were significantly reduced in all exposed animals. A normocytic anaemia was present in ETU treated animals; whereas normocytic hypochromic anaemia was evident in CPF and co-exposed mice, also exhibiting a reduced reticulocyte count *vs* controls. All treated animals had a significant reduction in total white cell count *vs* controls, involving lymphocytes in all of them, monocytes in ETU and co-exposed mice and, finally, neutrophils only in co-treatment condition. A significant reduction in platelet count was present in all treated animals, reaching the lowest levels in co-treated mice. Overall, the exposure to ETU and CPF induced pancytopenia, with a more severe profile when molecules were co-administrated. It has been previously shown that the deletion of *Zfp36l2* induced pancytopenia and impaired erythropoiesis^30^,^31^. Since we did not obtain enough sorted erythroid precursors for RT-PCR analysis, we validated its expression together with *Egr1* transcript in the spleen that contained modest levels of erythropoiesis (data not shown). *Zfp36l2* was significantly inhibited in the co-exposure conditions and in the single treatments by RT-qPCR (Fig. 4D). Although decreased, the inhibition of *Egr1* transcript was not statistically significant (Fig. 4D).

The analyses of bone marrow evidenced a marked reduction of the erythroblast mass in co-exposed mice *vs* controls (Fig. 4E). The morphologic analysis of erythroid precursors revealed signs of dyserythropoiesis and erythrophagocytosis in co-treated mice (Supplementary Fig. 2D). The profile of erythroblast maturation showed an increased number of basophilic erythroblasts (Pop II) and a reduction in orthochromatic erythroblasts (Pop IV) (Fig. 4F), the latter displaying increased apoptosis (Fig. 4G). Thus, the co-exposure to CPF and ETU induced dyserythropoiesis and ineffective erythropoiesis characterized by a block in cell maturation and increased apoptosis in the late phase of erythropoiesis. The analyses of cell cycle for the different subpopulations revealed a general trend of cells to accumulate in G0/G1 phase with a reduction of cells in the S phase, reaching statistical significance only in polychromatic erythroblasts (Fig. 4H).

Collectively our results confirmed that *in vitro* toxicogenomics could predict cell type unrelated outcomes, their molecular mechanisms and markers *in vivo*.

## Discussion

In a complex scenario involving cells physiologically adopting compensation processes, do the *in vitro* and *in vivo* toxicogenomics have the same predictive strength in revealing biomarkers or unpredicted outcomes of exposure valid in animals and, hopefully, in humans ? This answer is pivotal in transforming the Tox21c suggestion into a methodological approach in chemical testing.

Moving from our experience^14^,^32^,^33^, we planned an *in vitro* toxicogenomics experiment, in differentiated cells (immortalized thyrocytes), to identify gene signatures, mechanisms of toxicity or/and adverse outcomes potentially active *in vivo* of two pesticides, ETU and CPF. They were chosen for their known effects on thyroid physiology^10^,^11^, up to now not characterized at the level of thyroid gene expression.

As already reported, the first level of *in vitro* toxicogenomics data analysis, conducted in terms of “hits”, distinguished the compounds, their dose and mixtures. Some transcripts and mechanisms of toxicity common to CPF and ETU were identified and verified *in vitro* and *in vivo.* Thus, CPF could be molecularly classified as THDC as inhibiting *Egr1*, *Hmga1* and *Zfp36l2.* These genes were not deregulated in the same cells exposed to BPA^14^. Therefore, the gene signature determined *in vitro* differentiated pesticides from BPA. The CPF and ETU signatures were not completely specific *in vivo*. In particular, some CPF DEGs were deregulated also in animals treated with ETU and vice versa. This happened because in the toxicogenomic analysis we applied a FC ≥ 2 as cut off excluding from the signature the genes deregulated less than 2 times, although statistically significant. Our data suggest that a better specificity could be reached through an all-encompassing approach in data analyses, using gene lists also including poorly regulated genes, that should be joined to the cross-platform integration of the omics data proposed for the classification of non-genotoxic compounds^34^. We considered a broad approach critical for organs subject to strong compensation processes, i.e. thyroid, because we expected that the full correspondence between doses and *in vitro*/*in vivo* effects could not be easy to obtain. Indeed in a previous study assessing the *in vivo* relevance of *in vitro*-obtained data for genotoxic carcinogens on hepatocytes, the correspondence between *in vitro* and *in vivo* results was obtained only for two out of three tested compounds^35^. Consequently, we conceived a multi-dose testing and a subsequent inclusive analysis of *in vitro* data as well as the *in vivo* evaluation of the effects of different doses and mixtures at different exposure times. Indeed, the *in vitro* results were confirmed *in vivo* only in some exposure conditions at longer exposure (PND 360), strengthening the need to overcome compensation processes not developed *in vitro*. We suggest that both aspects, multiple doses and long exposure windows, should be considered in planning experiments to investigate the causal link between high-throughput data produced *in vitro* and effects *in vivo*, not easily established till now. Notably, *in vitro* toxicogenomics can reduce the time required for a first evaluation of different classes of compounds, including THDCs.

We were surprised that no thyroid specific gene/pathway was revealed *in vitro* although we found reduced levels of FT4 in blood and of the thyroid specific *Tg* transcript. We noticed that IPA predicted several shared biofunctions, cell cycle, etc., as mechanism of toxicity for endocrine system, identified by *Egr1* and *Hmga1.* These genes are known players of cellular proliferation in thyrocytes or other cell types^23^,^24^. We showed that both pesticides increased the apoptosis *in vivo* (inhibition of *Bcl2* transcript) that could be responsible for the decrease in circulating FT4. These results are in agreement with previous studies showing a higher number of pyknotic nuclei and dead cells in the thyroid follicular epithelium of rodents exposed to ETU^11^ and CPF^10^ associated to low levels of serum thyroid hormones. Furthermore, our data provide candidate molecular mechanisms (deregulated expression of *Bcl2, Zfp36l2*, *Egr1* and *Hmga1*) for the pesticides-induced hypothyroidism. Despite the fact that the weight of evidence (WoE) evaluation for chlorpyrifos evidenced no potential interaction with thyroid pathways at doses below the ones inhibiting the cholinesterase^36^, our findings are in agreement with epidemiological studies. Indeed, impairment of thyroid activity has been described among farmers using mancozeb (of which the ETU is the metabolite)^37^, among orgonophosphate pesticide formulators (CPF)^38^, and among men who were partners in subfertile couples and having high urine levels of 3,5,6-trichloro-2-pyridinol (TCPY), a metabolite of chlorpyrifos^39^. Our transcriptomic IPA analysis drove towards the characterization of the response to proliferation stimuli of the pesticides-treated thyrocytes *in vitro*, suggesting the molecular involvement of *Egr1* and *Hmga1* in the observed phenotype. This alteration of cell proliferation was revealed clearly after deprivation from TSH, mimicking an adverse condition that could be modelled *in vivo* by the co-exposure conditions. These data suggest that *in vivo* phenotypic validation of *in vitro* molecular data should be conducted considering that, in a real scenario, different insults can synergize lowering the adaptation response and producing, finally, phenotypic damages.

Our work evidenced the power of *in vitro* toxicogenomics in predicting pesticide effects not related to thyrocyte biology such as damages in erythropoiesis. Indeed the bioinformatic prediction obtained on thyroid cells resulted to be relevant when assessing the erythropoietic function *in vivo*. In our experimental settings the exposure to CPF or ETU and to their mixture reduced the number of mature white and red blood cells. Specifically, the reduction in circulating erythrocytes was due to a block in cell maturation and increased apoptosis in the late phase of erythropoiesis. Although in our experiment we only analysed the erythroblast populations, a recent publication demonstrated defective maturation of different marrow cell lineages due to a block in cell proliferation and increased apoptosis of hematopoietic precursor cells following exposure to a mixture of pesticides (including chlorpyrifos). The pancytopenia observed in the exposed mice was linked to defects of the bone marrow stromal cells^29^. In addition the exposure of hematopoietic stem cells from human cord blood to mancozeb reduced the clonogenic potential of erythroid and granulocyte-macrophage progenitors^40^. Here, we confirmed these effects in a realistic exposure scenario and we propose a molecular mechanism behind the observed phenotype consisting in the inhibition of *Egr1*, *Hmga1* and *Zfp36l2* transcripts. These genes are evidently part of complex and different gene networks regulating cell cycle/apoptosis in the thyroid as well as in the hematopoietic system.

In conclusion, the *in vitro* transcriptomic can support the identification of gene signatures, toxicity mechanisms and adverse effects also active *in vivo*. Although lacking in specificity, it shows a good predictive strength for mechanisms involving broad cell functions (i.e. cell cycle regulation, apoptosis) also when compensation processes are possible. Furthermore, the *in vitro* prediction of unexpected outcomes could allow a better planning for *in vivo* experiments, matching the 3 R rules.

Overall the presented data highlight the strength and limits of the Tox21c vision pointing to experimental approaches that can be undertaken to improve both *in vitro* toxicogenomics testing and *in vivo* phenotypic validation in order to establish cause-effect links. This is pivotal to transform the Tox21c suggestion into a real strategy in chemical testing.

## Methods

### Cell culture and treatment

PCCl3 were maintained in Coon’s modified F12 medium (EuroClone) supplemented with 5% newborn bovine serum (HyClone Laboratories) and six hormones^16^. Cells were exposed for 7 days to CPF (6 × 10^−7^ M, 6 × 10^−8^ M, 6 × 10^−9^ M, Greyhound Chromatography F2057) and ETU (6 × 10^−8^ M, 6 × 10^−9^ M, 6 × 10^−10^ M, 2-Imidazolidinethione, Sigma-Aldrich I504) (Table 1) and their combinations. Cell viability was analysed by MTT assay^13^. Briefly, 1 × 10^3^ cells were plated in 96-well plates and treated for 7 days. After 3-day long TSH deprivation, TSH was added and proliferation assessed at 24, 48 and 72 hrs.

### Animals and treatments

Animal experiments were performed in accordance with the European Council Directive 86/609/EEC following the rules of the D.Lvo 116/92 (ID number 25–10) and procedures were approved by the Ethical committee named CESA (Committee for the Ethics of the Experimentations on Animals) of the Biogem Institute of Genetics Research “Gaetano Salvatore” (IRGS). Mice were kept under standard facility conditions and received water and standard diet (4RF21, Mucedola) “ad libitum”. CD1 dams (outbred strain, 8 mice/treatment group) were exposed, 7 days before the mating, to pesticides dosed at 10 mg/kg/day, 1 mg/kg/day, 0.1 mg/kg/day, and the combinations of higher and lower doses, administrating ETU by drinking water (59, 5.9 and 0.59 mg/L), and CPF by food at 44, 4.4 and 0.44 mg/kg (Mucedola) till the weaning. Therefore, the offspring were exposed through the mothers from gestational day 0 (GD0) till the weaning. Then, the offspring (10 females and males) were directly exposed. Animals were sacrificed at 6 months, 4 mice/group, and at 12 months, 6 mice/group, for blood and organs collection, by carbon dioxide inhalation.

### RNA extraction, sequencing and RT-qPCR

RNA was prepared as already reported^13^. For RNA-seq samples were treated with DNasi (NEB), purified with RNasy Mini Kit (Qiagen) and controlled with Agilent 2100 bioanalyzer (Agilent Technologies). RNAs, 3 μg/each obtained from three biological replicates, were pooled to minimize sample-to-sample variations. 1 μg of each RNA pool was used for library preparation with TruSeq Stranded total RNA Sample Prep Kit (Illumina Inc.) according to the manufacturer’s instructions. Libraries were sequenced in triplicates (paired-end, 2 × 100 cycles) at a concentration of 8 pmol/L per lane on HiSeq2500 platform (Illumina Inc.).

The generated raw sequence files (.fastq files) underwent quality control analysis using FastQC (http://www.bioinformatics.babraham.ac.uk/projects/fastqc/) and will be available at the ArrayExpress database (http://www.ebi.ac.uk/arrayexpress.).

RT-qPCR and primer design were conducted as already described^13^ using QuantiTect Reverse Transcription Kit (QIAGEN), Power SYBR Green Master Mix (Applied Biosystems with Applied Biosystem 7900 Real-Time PCR System) and NCBI Primer Blast, respectively. See Supplementary Table S1 for primer sequences. The expression values were normalized on the relative expression of *Gapdh*.

### RNA sequencing data and bioinformatics analyses

For RNA seq data analysis about 3 × 10^7^ high quality sequences reads pairs were produced for each sample. The rat UCSC rn5 genome was used as reference for read mapping, by TopHat aligner, further processed using Cufflinks package^41^. Normalized counts were represented by a box plot across the conditions to assess the quality of processed data (see Fig. S1). Selected reads were assembled in transcripts, compared among the samples and the unique final transcriptome generated. Differential expression was tested for the resulting 23762 genes comparing each pesticide treatment *vs* control. Significantly deregulated genes were selected by corrected p-value (FDR) ≤ 0.05 and absolute FC ≥ 2. Genes significantly deregulated at least in one dose were partitioned in three different sets through K-means clustering analysis using Jensen-Shannon (JS) distance as metric^22^.

Assembled rat genes without annotation were compared with the human genome to find their putative orthologous, using UCSC xenoRefGene table. The human gene with highest base identity was proposed to annotate the corresponding rat gene.

Functional enrichment analysis was performed using IPA (http://www.ingenuity.com). Over-represented biological and toxicological functions were found by Fisher exact test. Significant categories were selected by B–H corrected p-value ≤ 0.05.

### FT4 determination, blood and bone marrow analysis

The serum FT4 level was determined with the FT4 ELISA kit (Diametra) following the manufacturer instructions.

Hematological parameters and red cell indices were evaluated with ADVIA 2120 Hematology System (Siemens Healthcare GmbH). Hematocrit and hemoglobin were manually determined^42^. Flow cytometric analysis of erythroid precursors from bone marrow was carried out using the CD44-TER-119 using the FACSCanto IITM flow cytometer (Becton Dickinson). Population II, III and IV were sorted from bone marrow, as previously reported^43^. Morphological analysis of sorted erythroblasts was performed on cytospin preparations stained with May Grunwald-Giemsa. Apoptosis in orthochromatic erythroblasts was carried out on CD44-Ter-119 gated cells (Annexin-V PE Apoptosis detection kit, eBioscience,). Cell cycle analysis was carried out by DAPI staining and data analyzed with FlowJo (Tree Star, Ashland) using Watson model.

### Statistical and bioinformatics analyses

Statistical analyses were performed using Student’s t-test. Probability *p-*values below 0.05 were considered significant. *, **, ***Indicate *p-*value < 0.05, <0.01 and <0.001, respectively. Unless otherwise indicated, at least three independent experiments were considered for *in vitro* data and four animals for in *vivo* experiments. The results are expressed as the mean ± standard deviation.

## Additional Information

**How to cite this article**: Porreca, I. *et al*. Pesticide toxicogenomics across scales: *in vitro* transcriptome predicts mechanisms and outcomes of exposure *in vivo*. *Sci. Rep.* **6**, 38131; doi: 10.1038/srep38131 (2016).

**Publisher's note:** Springer Nature remains neutral with regard to jurisdictional claims in published maps and institutional affiliations.

## Supplementary Material

Supplementary Information

## Figures and Tables

**Table 1:** ETU and CPF applied concentrations *in vitro*.

	*In vitro* applied concentration	Abbreviation
CPF-high dose	6 × 10^−7 ^M	CPF-H
CPF-medium dose	6 × 10^−8 ^M	CPF-M
CPF- low dose	6 × 10^−9 ^M	CPF-L
ETU-high dose	6 × 10^−8 ^M	ETU-H
ETU-medium dose	6 × 10^−9 ^M	ETU-M
ETU- low dose	6 × 10^−10 ^M	ETU-L

**Table 2:** *In vivo* validation of thyroid toxicity signatures at PND360.

	Gene name	ETU mg/kg/day			CPF mg/kg/day			ETU + CPF mg/kg/day	
		0.1	1	10	0.1	1	10	0.1	10
*1*	*Zfp36l2*	−1.51	−1.99*	−2.17**	−1.14	−1.83*	−1.49	−1.78*	−3.14**
*2*	*Hmga1*	1.3	−1.07	−1.42*	−1.05	−1.14	−1.27	−1.34	−1.15
*3*	*Egr1*	1.49	−1.41	−2.6**	−1.29	−1.88*	−1.59	−1.59	−2.6*
*4*	*Ergic1*	−1.2	−1.3	−1.47*	−1.12	−1.16	−1.19	−1.23	−1.59
*5*	*Zfp524*	−1.67	1.01	−1.4*	1.31	1.01	1.11	1.04	−1.11
*6*	*Ifit3*	−1.61	−1.17	−2.68	1.04	1.05	−1.12	−2.14	−1.01
*7*	*Fz5*	−2.55*	−2.8**	−4.6**	−1.2	−3.04**	−3.24**	−2.01*	−2.27
*8*	*Gnao1*	−1.77	−2.03	−2.04	−1.6	−1.57	−2.16*	−1.93	−1.32
*9*	*Runx2*	1.1	1	−1.51	1.4	−1.21	−1.13	−1.1	−1.26

Transcript level of genes in common- (1–3), ETU- (4–6) and CPF- (7–9) signatures was validated by RT-qPCR. Data are reported as FC *vs* controls. In bold the ones reaching statistical significance.

**Table 3:** Hematological parameters and red cell indices at PND 360.

	Ctrl (*n* = 10)	ETU (0.1 mg/kg/day) (*n* = 4)	CPF (0.1 mg/kg/day) (*n* = 4)	ETU + CPF (0.1 mg/kg/day) (*n* = 10)
Hct (%)	47.2 ± 1.08	42.6 ± 2.2*	41.0 ± 2.6*	40.3 ± 1.6*
Hb (g/dl)	15.6 ± 0.7	13.8 ± 1.2*	13.6 ± 0.9*	13.2 ± 0.4*
MCV (fl)	53.4 ± 1.0	52.7 ± 1.1	52.3 ± 0.5	52.7 ± 0.7
MCH (g/dl)	16.7 ± 1.5	15.0 ± 0.7	14.8 ± 2.6*	14.1 ± 0.5*
Retics (10^3^ cells/μL)	194 ± 48	135 ± 24	113 ± 15	73.9 ± 19*
WBC (cells/μL)	1330 ± 324	450 ± 278*	780 ± 451*	862 ± 181*
N (cells/μL)	378 ± 115	134 ± 69	273 ± 108	**157 ± 27**
L (cells/μL)	540 ± 279	240 ± 131	321 ± 158	433 ± 95
M (cells/μL)	16 ± 7.8	2.52 ± 0.9*	14.8 ± 9.4	4.2 ± 0.1*
PLTs (10^3^ cells/μL)	847 ± 12	743 ± 5	731 ± 11	169 ± 9.0*

**Hct:** hematocrit; **Hb:** hemoglobin; **MCV:** mean corpuscular volume; **MCH:** mean corpuscular hemoglobin; **Retics:** reticulocytes; **MCVr:** mean corpuscular volume reticulocytes; **WBC:** white blood cells; **N:** neutrophil**; L:** lymphocyte**; M:** monocyte; **PLTs:** platelets. Data are reported as means ± SD. In bold the ones reaching statistical significance.
