# Supplementary material for: Pesticide toxicogenomics across scales: in vitro transcriptome predicts mechanisms and outcomes of exposure in vivo
Source: Sci Rep. 2016 Dec 1;6:38131. doi: 10.1038/srep38131 (PMC5131489; doi:10.1038/srep38131)
Supplement: Supplementary Information [file srep38131-s1.doc]

**Supplementary Material**

**Pesticide toxicogenomics across scales: *in vitro* transcriptome predicts mechanisms and outcomes of exposure *in vivo.***

Immacolata Porreca*^,1^, Fulvio D’Angelo*^,1^, Lucia De Franceschi^2^, Alessandro Mattè^2^, Michele Ceccarelli^3^, Achille Iolascon^4^, Alberto Zamò^5^, Filomena Russo^1^, Maria Ravo^6^, Roberta Tarallo^6^, Marzia Scarfò^1^, Alessandro Weisz^6^, Mario De Felice^7^, Massimo Mallardo^§,4^ and Concetta Ambrosino^§,1,3^.

^1^IRGS, Biogem, Via Camporeale, 83031, Ariano Irpino, Avellino, Italy

^2^Department of Medicine, University of Verona-AOUI Verona, Policlinico GB Rossi; P.Le L. Scuro, 10; 37134 Verona, Italy

^3^Department of Science and Technology, University of Sannio, Via Port’Arsa 11, 82100, Benevento, Italy

^4^Molecular Medicine and Medical Biotechnologies, University of Naples “Federico II Napoli, Italy

^5^Department of Diagnostics and Public Health, University of Verona-AOUI Verona, Policlinico GB Rossi; P.Le L. Scuro, 10; 37134 Verona, Italy

^6^Laboratory of Molecular Medicine and Genomics, Department of Medicine, Surgery and Dentistry ‘Schola Medica Salernitana’, University of Salerno, Baronissi, Salerno, Italy

^7^IEOS-CNR, Via Pansini 6, 80131 Napoli, Italy

^*^These authors contributed equally to this work.

Correspondence and requests for materials should be addressed to M.M. (email: mallardo@dbbm.unina.it) or to C. A. (email: coambros@unisannio.it).

**Supplementary Figures**


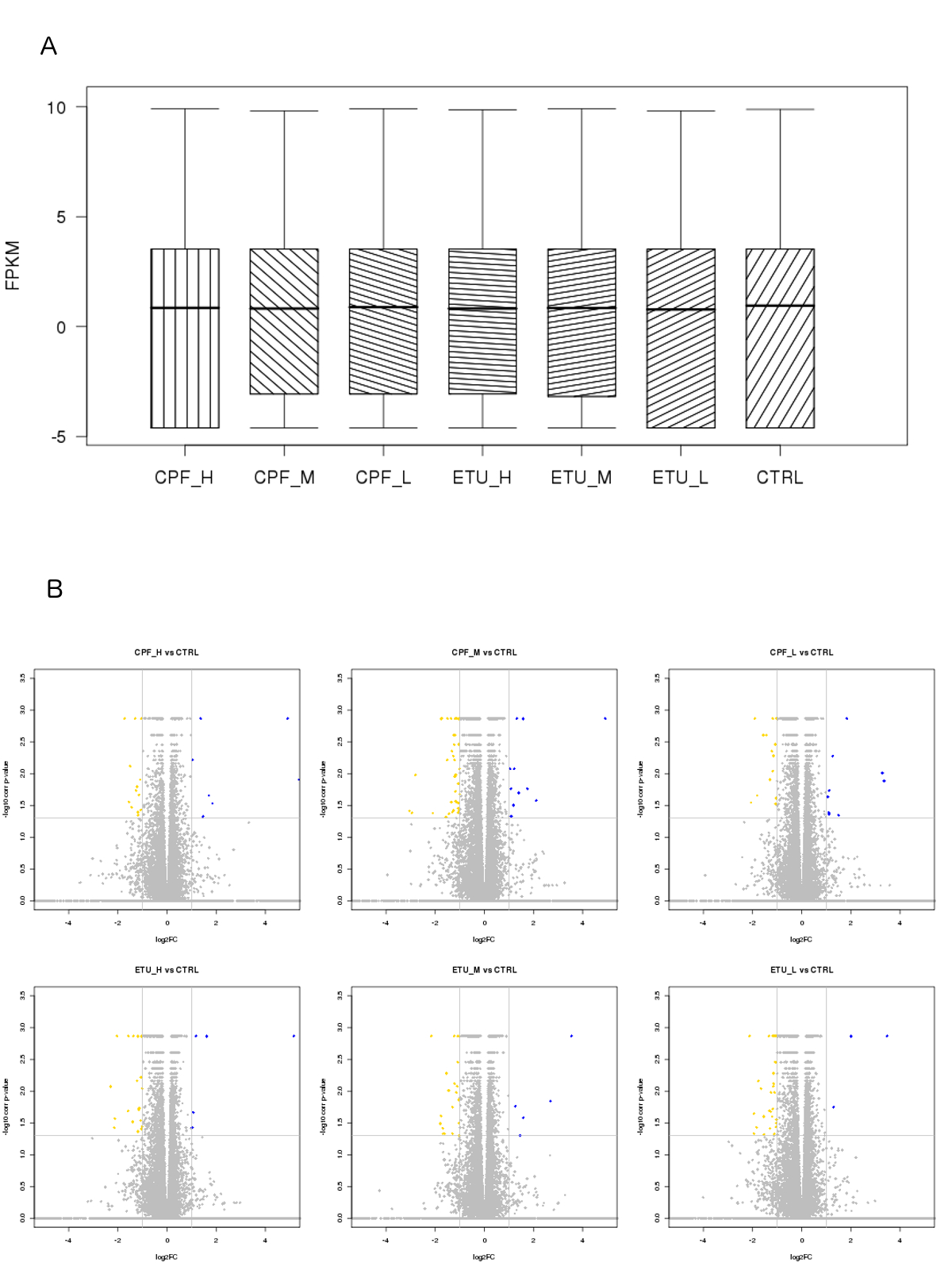


**Supplementary Figure 1 – Gene expression overview**

(A) Box-plot of FPKM normalized distributions, showing that the median value (black line) was the same across the samples. (B) Differential gene expression between each condition versus control was represented by Volcano plot. Significant genes are coloured in yellow (down-regulated) or in bleu (up-regulated).

**
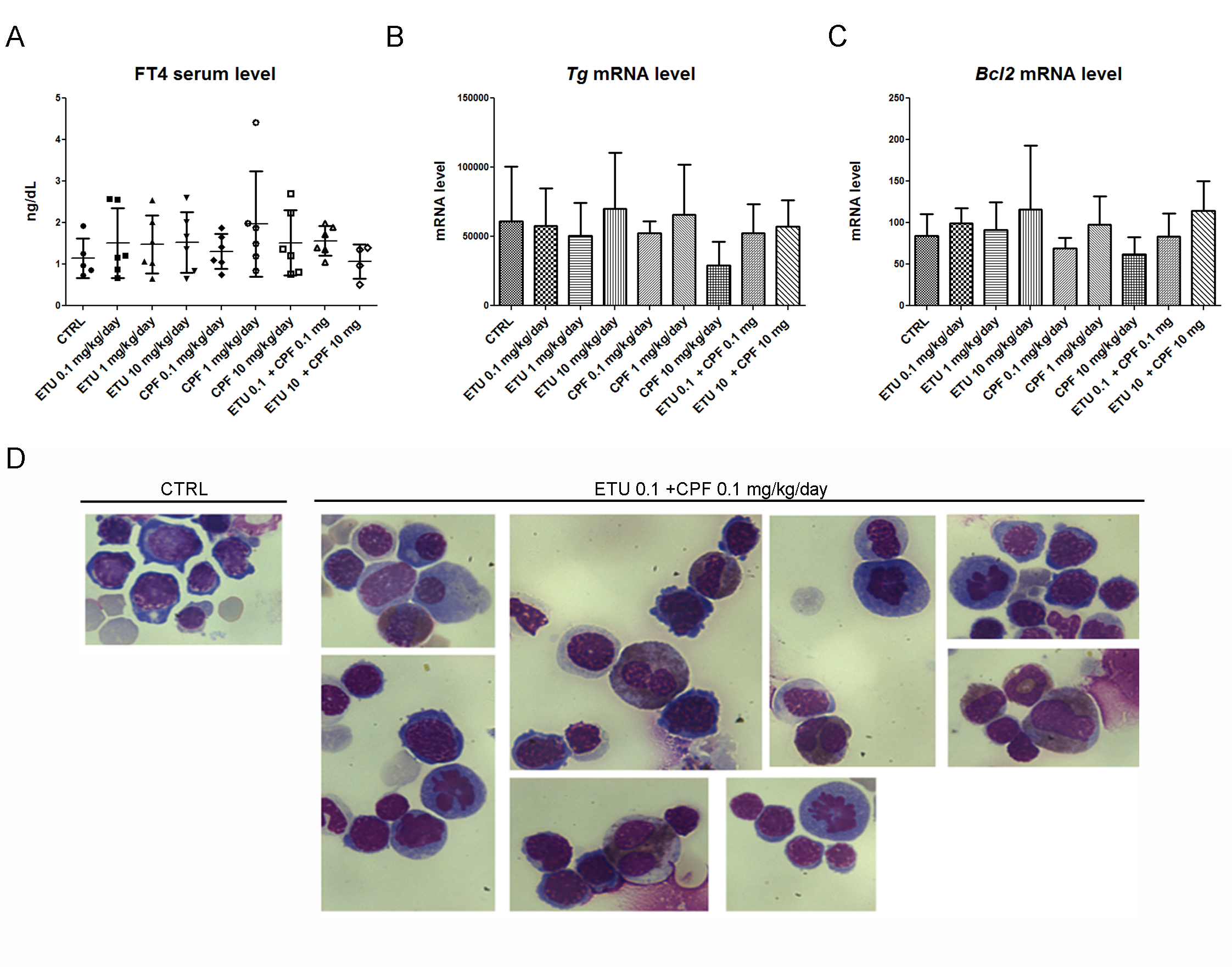
**

**Supplementary Figure 2** **– *In vivo* (PND 180) validation of the mechanisms of thyroid toxicity of CPF and ETU.** (A) FT4 serum level in females exposed to ETU or CPF (10, 1, 0,1 mg/kg/day) and their combination at 10mg/kg/day and 0,1 mg/kg/day. Each sign is a single mouse. Mean and standard deviation is reported. Level of *Tg* (B) and *Bcl2* (C*)* transcripts in thyroid of females exposed as above. Data are reported as the average and standard deviation of *Gapdh*-normalized mRNA levels of 4 animals. (D) Morphology of sorted erythroblasts. Cytospins were stained with May-Grunwald-Giemsa. Cells were imaged under oil at 100x magnification using a Panfluor objective with 1.30 numeric aperture on a Nikon Eclipse DS-5M camera and processed with Digital Slide (DS-L1) Nikon. One representative image from a total of 10 for each mouse groups is reported.

**
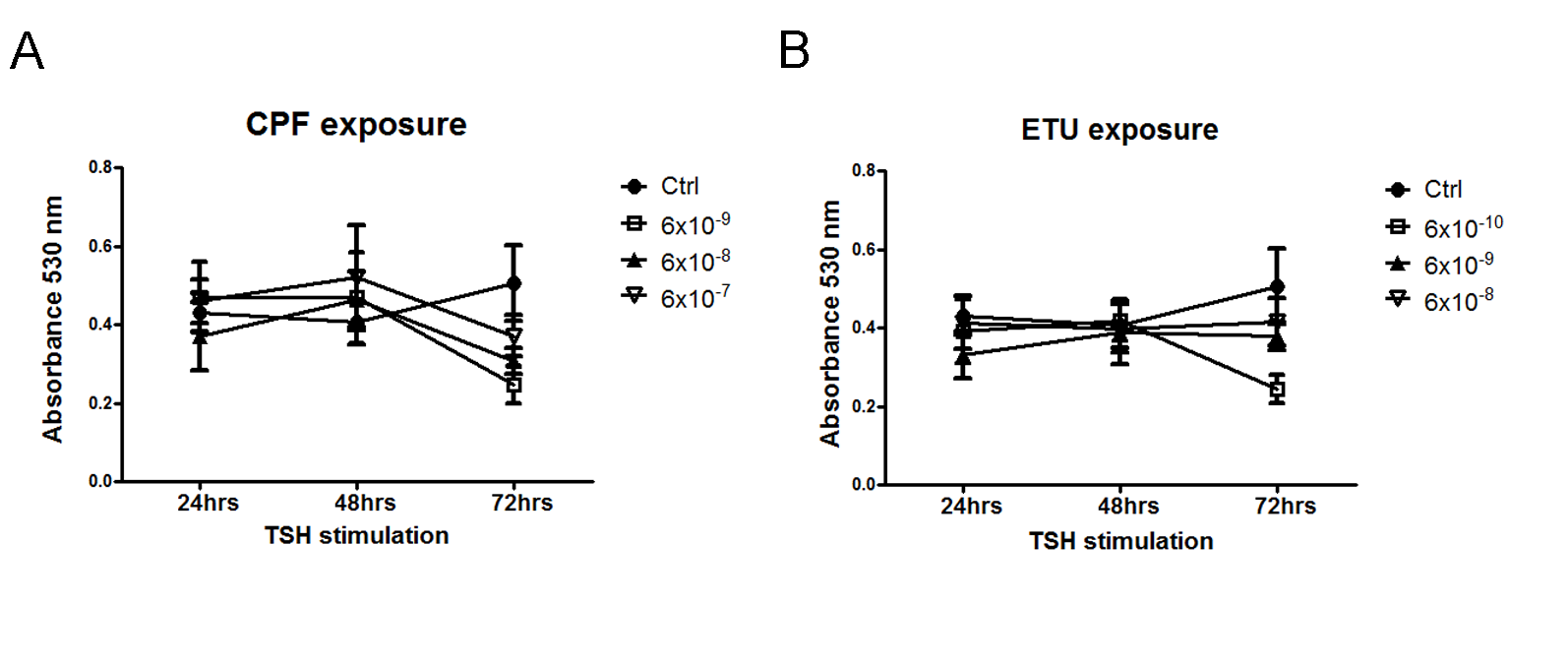
**

**Supplementary Figure 3** ***In vitro* validation of cell cycle alteration.** MTT assay on PCCl3 cells treated with CPF (A) or ETU (B) at the reported concentrations, treated with TSH for 24, 48, 72 hrs, after its deprivation for 3 days.

**Supplementary Table 1** CPF cluster #1 genes.

Differential expression of cluster genes is reported as log2FoldChange between treated vs control cells; significant values (FDR≤0.05) are represented in bold.

| **Gene ID** | **HumanAlignedGene** | **CPF-high** | **CPF-medium** | **CPF-low** | **CxE_high** | **CxE_medium** | **CxE_low** |
| --- | --- | --- | --- | --- | --- | --- | --- |
| **Bex2** |  | 0.46 | ***-2.81*** | ***-1.29*** | 0.03 | ***-3.07*** | -3.76 |
| **Ddit4** |  | 0.03 | ***-1.18*** | ***-0.81*** | ***-0.21*** | ***-0.73*** | ***-0.66*** |
| **Egr1** |  | -0.08 | ***-0.71*** | ***-1.02*** | ***-0.44*** | ***-0.71*** | ***-0.63*** |
| **Fscn2** |  | -0.58 | -1.14 | ***-1.04*** | -0.70 | -0.79 | ***-1.13*** |
| **Fus** |  | -0.08 | ***-1.11*** | ***-0.97*** | ***-0.63*** | -0.16 | -0.05 |
| **Fzd5** |  | -0.35 | ***-1.03*** | ***-0.79*** | -0.32 | ***-0.51*** | ***-0.63*** |
| **Gnao1** |  | -0.38 | ***-1.04*** | ***-1.18*** | ***-0.57*** | ***-0.60*** | ***-0.46*** |
| **Gtf3c6** |  | ***-1.04*** | ***-0.20*** | ***0.34*** | ***-0.16*** | ***-0.54*** | ***-1.06*** |
| **Hexim2** |  | -0.34 | -0.75 | ***-1.15*** | -0.39 | 0.97 | -0.15 |
| **Hmga1** |  | -0.74 | ***-1.17*** | ***-1.44*** | -0.72 | ***-1.45*** | -1.14 |
| **Inpp5j** |  | -0.50 | ***-1.12*** | ***-0.83*** | ***-0.67*** | ***-0.82*** | ***-0.77*** |
| **Mir3564** |  | ***5.36*** | -0.64 | -0.85 | -1.30 | -0.53 | -1.05 |
| **Pak2** |  | 0.49 | ***-1.16*** | -0.53 | 0.32 | ***-1.11*** | ***-1.75*** |
| **Reep6** |  | ***-0.43*** | ***-1.00*** | ***-0.79*** | ***-0.88*** | ***-0.99*** | ***-0.63*** |
| **RGD1304978** |  | ***-1.50*** | -0.37 | -0.56 | -0.58 | -0.27 | -0.21 |
| **Runx2** |  | -0.41 | ***-0.92*** | ***-1.08*** | -0.75 | -1.04 | ***-1.16*** |
| **Zfp362** |  | -0.68 | ***-1.06*** | ***-1.00*** | ***-1.06*** | ***-0.89*** | -0.73 |
| **Zfp36l2** |  | 0.09 | ***-1.51*** | ***-1.56*** | -0.29 | -2.13 | -2.44 |
| **Zfp703** |  | -0.04 | ***-1.18*** | ***-0.96*** | -0.63 | -0.85 | -0.85 |
| XLOC_000226 | ZNF628 | -0.24 | ***-0.85*** | ***-1.01*** | ***-0.53*** | ***-1.16*** | -0.44 |
| XLOC_000904 | DMBT1, DMBT1P1 | -0.71 | ***-1.03*** | ***-1.03*** | ***-0.85*** | ***-1.12*** | ***-0.95*** |
| XLOC_001006 |  | -1.15 | ***-1.56*** | -1.70 | -0.88 | -0.42 | -1.09 |
| XLOC_001830 | PRR12, PRRG2 | -0.25 | ***-1.13*** | ***-0.69*** | ***-0.93*** | -0.59 | ***-0.74*** |
| XLOC_001996 |  | 0.04 | ***-1.03*** | -0.66 | -0.50 | ***-1.00*** | ***-0.92*** |
| XLOC_002795 |  | -0.48 | ***-1.23*** | -0.64 | -0.49 | ***-0.82*** | ***-0.97*** |
| XLOC_002799 |  | -0.26 | ***-1.07*** | -0.46 | -0.22 | -0.46 | -0.45 |
| XLOC_002804 |  | ***-0.56*** | ***-1.19*** | ***-0.75*** | ***-0.57*** | ***-0.57*** | ***-0.80*** |
| XLOC_004999 | CLEC16A | 0.17 | ***-1.07*** | -0.47 | -0.61 | -0.59 | -0.89 |
| XLOC_005067 |  | -0.17 | -0.56 | ***-1.06*** | -0.43 | -0.68 | -0.28 |
| XLOC_005096 |  | ***-1.13*** | -0.77 | -0.52 | -0.19 | -0.36 | -0.71 |
| XLOC_006469 |  | -0.68 | ***-1.13*** | -0.57 | -0.83 | ***-1.11*** | -0.63 |
| XLOC_007262 | SMG7 | -0.64 | ***-1.26*** | -0.52 | ***-0.77*** | -0.32 | -0.50 |
| XLOC_007929 |  | -0.34 | ***-1.01*** | ***-1.21*** | -0.36 | -0.57 | -0.50 |
| XLOC_007983 | NWD2 | ***-1.19*** | -0.99 | -1.07 | -0.68 | -0.56 | ***-1.07*** |
| XLOC_008002 | CPEB2 | -0.46 | ***-1.00*** | -0.67 | -0.50 | -0.23 | -0.56 |
| XLOC_009258 |  | ***-1.55*** | -1.06 | -0.57 | -0.43 | -0.33 | -0.90 |
| XLOC_009994 | KIAA1217 | ***-1.17*** | -0.42 | -0.24 | -0.03 | -0.17 | -0.31 |
| XLOC_010335 | SEMA6A | -0.56 | ***-1.06*** | -0.69 | -0.51 | ***-1.33*** | -0.57 |
| XLOC_010494 | CHSY3 | -0.83 | ***-1.05*** | -0.49 | -0.31 | -0.32 | -0.89 |
| XLOC_010499 | CHSY3 | -0.71 | ***-1.30*** | -0.66 | -0.57 | -0.46 | -0.86 |
| XLOC_010502 | CHSY3 | -0.81 | ***-1.36*** | -0.66 | -0.63 | -0.13 | -0.23 |
| XLOC_011115 |  | 0.05 | ***-1.73*** | ***-1.92*** | ***-0.89*** | ***-0.87*** | ***0.70*** |
| XLOC_011123 | CHD9 | ***-1.22*** | ***-1.17*** | -0.45 | ***-1.47*** | -0.78 | -0.77 |
| XLOC_011155 | SLC25A36 | -0.69 | ***-1.07*** | -0.43 | ***-0.72*** | -0.46 | -0.55 |
| XLOC_011215 |  | ***-1.73*** | ***-1.50*** | ***0.48*** | ***-0.84*** | ***-3.20*** | ***-2.06*** |
| XLOC_011228 |  | ***0.94*** | ***-1.06*** | 0.57 | ***0.85*** | ***-1.54*** | ***-2.60*** |
| XLOC_011229 | TARBP1 | -1.11 | ***-2.10*** | -1.05 | -1.18 | -0.64 | -1.14 |
| XLOC_014273 | C9orf172 | ***-0.78*** | ***-1.21*** | ***-1.18*** | ***-0.74*** | ***-1.16*** | ***-0.91*** |
| XLOC_015271 | WDR76, FRMD5 | -0.61 | ***-1.35*** | -0.46 | -0.40 | -0.13 | -0.83 |
| XLOC_015275 | WDR76, FRMD5 | ***-0.70*** | ***-1.14*** | -0.45 | ***-0.88*** | ***-0.58*** | ***-0.62*** |
| XLOC_015408 |  | -0.86 | ***-1.44*** | -1.32 | -0.77 | -0.63 | ***-1.56*** |
| XLOC_017823 |  | ***-1.30*** | ***-0.20*** | ***0.17*** | ***0.96*** | ***0.21*** | ***-0.53*** |
| XLOC_018043 |  | -0.46 | ***-1.25*** | -0.86 | -0.55 | -0.40 | -0.26 |
| XLOC_018090 | CTNNAL1 | -0.39 | ***-1.32*** | -0.56 | ***-0.86*** | -0.25 | -0.53 |
| XLOC_018117 | DENND4C | ***-1.10*** | ***-1.23*** | ***-0.90*** | ***-0.84*** | ***-1.26*** | -0.46 |
| XLOC_018736 | CDKL4 | ***-4.03*** | 0.30 | -1.16 | -0.71 | -1.00 | -0.71 |
| XLOC_019172 |  | ***-1.44*** | ***-3.06*** | ***-1.82*** | ***-1.33*** | -0.84 | -0.84 |
| XLOC_019221 |  | -1.15 | ***-2.95*** | ***-2.06*** | ***-1.78*** | ***-1.11*** | -1.03 |
| XLOC_019237 | KIAA0247 | -0.75 | ***-1.20*** | -0.64 | -0.44 | -0.71 | -0.52 |
| XLOC_021936 |  | -0.51 | ***-1.04*** | -0.41 | -0.59 | ***-0.79*** | -0.54 |
| XLOC_021960 |  | ***-1.26*** | 0.02 | -0.75 | 0.27 | -0.26 | -0.36 |
| XLOC_022000 |  | -0.30 | ***-1.76*** | ***-0.83*** | -0.53 | -0.31 | -0.44 |

**Supplementary Table 2** ETU cluster #1 genes.

Differential expression of cluster genes is reported as log2FoldChange between treated vs control cells; significant values (FDR≤0.05) are represented in bold.

| **Gene ID** | **HumanAlignedGene** | **ETU-high** | **ETU-medium** | **ETU-low** | **CxE_high** | **CxE_medium** | **CxE_low** |
| --- | --- | --- | --- | --- | --- | --- | --- |
| **Actl7a** |  | ***-0.94*** | -0.46 | ***-1.10*** | -0.58 | -0.61 | -0.20 |
| **Bex2** |  | ***-2.30*** | -0.50 | ***-1.77*** | 0.03 | ***-3.07*** | -3.76 |
| **Ddit4** |  | ***-0.85*** | ***-1.09*** | ***-0.37*** | ***-0.21*** | ***-0.73*** | ***-0.66*** |
| **Egr1** |  | ***-1.18*** | ***-0.90*** | ***-0.64*** | ***-0.44*** | ***-0.71*** | ***-0.63*** |
| **Fam46d** |  | -0.23 | -0.51 | ***-1.03*** | -0.44 | ***-0.80*** | ***-0.76*** |
| **Fscn2** |  | ***-1.01*** | ***-1.05*** | ***-1.02*** | -0.70 | -0.79 | ***-1.13*** |
| **Fus** |  | ***-0.96*** | ***-1.23*** | ***-0.29*** | ***-0.63*** | -0.16 | -0.05 |
| **Hexim2** |  | ***-1.07*** | ***-1.55*** | -0.89 | -0.39 | 0.97 | -0.15 |
| **Hist3h2bb** |  | -0.72 | ***-1.68*** | -0.63 | 0.24 | -0.19 | -1.09 |
| **Hmga1** |  | ***-1.15*** | -0.83 | ***-1.17*** | -0.72 | ***-1.45*** | -1.14 |
| **Ifit3** |  | -0.44 | ***-1.79*** | -0.54 | -0.22 | -0.52 | -0.18 |
| **Pom121l2** |  | ***-0.90*** | ***-1.04*** | -0.56 | 0.27 | ***-0.92*** | ***-1.50*** |
| **Zfp36l2** |  | ***-2.03*** | ***-1.14*** | ***-1.14*** | -0.29 | -2.13 | -2.44 |
| **Zfp524** |  | ***-1.13*** | -0.53 | -0.19 | -0.29 | ***-0.77*** | -0.66 |
| **Zfp703** |  | ***-1.05*** | ***-0.77*** | ***-0.97*** | -0.63 | -0.85 | -0.85 |
| XLOC_000904 | DMBT1, DMBT1P1 | ***-1.04*** | ***-1.01*** | -0.75 | ***-0.85*** | ***-1.12*** | ***-0.95*** |
| XLOC_001477 | PDE10A | ***-0.71*** | -0.37 | ***-1.05*** | -0.44 | ***-0.68*** | ***-0.58*** |
| XLOC_001830 | PRR12, PRRG2 | ***-1.18*** | ***-0.95*** | ***-0.55*** | ***-0.93*** | -0.59 | ***-0.74*** |
| XLOC_002782 | SASH1 | ***-0.75*** | ***-0.67*** | ***-1.06*** | ***-0.56*** | ***-0.57*** | -0.37 |
| XLOC_002787 | SASH1 | ***-0.67*** | ***-0.66*** | ***-1.15*** | ***-0.49*** | ***-0.56*** | -0.38 |
| XLOC_002788 | SASH1 | -0.72 | -0.70 | ***-1.54*** | -0.60 | -0.29 | -0.58 |
| XLOC_002795 |  | ***-0.78*** | ***-1.09*** | ***-1.11*** | -0.49 | ***-0.82*** | ***-0.97*** |
| XLOC_002804 |  | ***-0.85*** | ***-0.88*** | ***-1.09*** | ***-0.57*** | ***-0.57*** | ***-0.80*** |
| XLOC_002812 |  | ***-1.40*** | -0.77 | ***-1.13*** | ***-1.15*** | -0.56 | -0.65 |
| XLOC_003085 | XRRA1, RNF169 | -0.99 | -0.78 | ***-1.95*** | -0.84 | -1.34 | -1.14 |
| XLOC_003288 | CPEB3 | -0.79 | -0.65 | ***-1.25*** | -0.51 | -0.56 | -0.40 |
| XLOC_003304 | LCOR, C10orf12 | ***-0.88*** | ***-0.83*** | ***-1.05*** | ***-0.72*** | ***-0.65*** | ***-0.69*** |
| XLOC_003403 |  | -0.89 | ***-1.02*** | -0.92 | ***-0.94*** | ***-0.93*** | ***-0.94*** |
| XLOC_005027 | ERGIC1 | ***-1.07*** | -0.46 | -0.38 | -0.61 | 0.11 | ***-1.16*** |
| XLOC_005133 |  | ***-0.94*** | ***-1.01*** | ***-0.64*** | ***-0.37*** | ***-0.17*** | -0.03 |
| XLOC_006469 |  | ***-1.04*** | ***-1.45*** | ***-1.65*** | -0.83 | ***-1.11*** | -0.63 |
| XLOC_007983 | NWD2 | -1.19 | ***-1.30*** | -0.48 | -0.68 | -0.56 | ***-1.07*** |
| XLOC_009126 |  | ***-0.58*** | ***-0.55*** | ***-1.09*** | -0.37 | -0.12 | -0.29 |
| XLOC_009258 |  | -0.72 | -0.97 | ***-1.29*** | -0.43 | -0.33 | -0.90 |
| XLOC_009979 |  | -0.72 | ***-1.62*** | -0.24 | -0.06 | -0.71 | -4.02 |
| XLOC_010009 |  | -0.83 | -0.90 | ***-1.31*** | -0.49 | -0.73 | -0.59 |
| XLOC_011115 |  | ***-1.57*** | ***-2.17*** | ***-0.60*** | ***-0.89*** | ***-0.87*** | ***0.70*** |
| XLOC_011123 | CHD9 | -1.13 | ***-1.29*** | -1.22 | ***-1.47*** | -0.78 | -0.77 |
| XLOC_011215 |  | ***-1.37*** | ***-0.94*** | ***-2.13*** | ***-0.84*** | ***-3.20*** | ***-2.06*** |
| XLOC_014273 | C9orf172 | ***-1.22*** | ***-1.11*** | ***-0.78*** | ***-0.74*** | ***-1.16*** | ***-0.91*** |
| XLOC_015117 | GAPVD1 | -1.01 | -0.90 | ***-1.95*** | -1.12 | -0.79 | -0.94 |
| XLOC_015175 |  | ***-0.85*** | ***-0.80*** | ***-1.01*** | ***-0.61*** | ***-0.61*** | ***-0.74*** |
| XLOC_015275 | WDR76, FRMD5 | ***-0.74*** | ***-0.84*** | ***-1.34*** | ***-0.88*** | ***-0.58*** | ***-0.62*** |
| XLOC_015302 | BTBD3 | ***-0.89*** | -0.69 | ***-1.17*** | -0.54 | ***-0.69*** | ***-0.81*** |
| XLOC_016752 | BOLA3, TET3 | -0.77 | -0.68 | ***-1.05*** | -0.60 | -0.45 | -1.08 |
| XLOC_017102 | TRABD2B, TRABD2A | ***-1.18*** | -0.49 | ***-0.85*** | -0.14 | -0.42 | ***-1.34*** |
| XLOC_018018 |  | -0.69 | -0.56 | ***-1.15*** | -0.58 | -0.31 | -0.53 |
| XLOC_018090 | CTNNAL1 | -0.69 | ***-1.21*** | ***-0.90*** | ***-0.86*** | -0.25 | -0.53 |
| XLOC_018117 | DENND4C | ***-0.89*** | -0.75 | ***-1.16*** | ***-0.84*** | ***-1.26*** | -0.46 |
| XLOC_019155 |  | ***-4.05*** | -1.82 | -1.51 | -1.74 | -10.60 | -2.83 |
| XLOC_019172 |  | ***-1.59*** | ***-1.77*** | ***-1.54*** | ***-1.33*** | -0.84 | -0.84 |
| XLOC_019221 |  | ***-2.13*** | ***-1.81*** | -2.84 | ***-1.78*** | ***-1.11*** | -1.03 |
| XLOC_019238 | KIAA0247 | -0.63 | ***-1.02*** | ***-1.13*** | -0.48 | -0.71 | -0.53 |
| XLOC_020614 | PHF20L1 | -0.50 | -0.62 | ***-1.26*** | -0.23 | -0.53 | -0.61 |
| XLOC_020648 | SGSM3, MKL1 | -0.71 | -0.66 | ***-1.51*** | -0.75 | -0.59 | -0.50 |
| XLOC_021936 |  | ***-1.01*** | -0.61 | ***-1.20*** | -0.59 | ***-0.79*** | -0.54 |
| XLOC_022000 |  | ***-1.02*** | ***-0.95*** | ***-0.97*** | -0.53 | -0.31 | -0.44 |
| XLOC_022034 | ZBTB38 | ***-0.87*** | ***-0.77*** | ***-1.07*** | ***-0.56*** | ***-0.74*** | ***-0.79*** |
| XLOC_023669 |  | ***-2.14*** | ***-1.70*** | ***-1.84*** | -1.26 | -1.41 | -1.19 |

**Supplementary Table 3** *In vivo* validation of thyroid toxicity signatures at PND180

|  | ETU mg/kg/day | | | CPF mg/kg/day | | | ETU+CPF mg/kg/day | |
| --- | --- | --- | --- | --- | --- | --- | --- | --- |
| Gene name | 0,1 | 1 | 10 | 0,1 | 1 | 10 | 0,1 | 10 |
| *Zfp36l2* | 1.01 | 1.31 | 1.03 | 1.5 |  | -1.21 | -1.24 | 1.48 |
| *Hmga1* | -1.08 | -1.33 | -1.13 | -1.49 |  | -1.86 | -2.31 | -1.5 |
| *Egr1* | 1.01 | -1.01 | -1.87 | -1.11 |  | 1.23 | -1.96 | -2.18 |

Transcript level of genes in common signatures was validated by RT-qPCR at PND 180. Data are reported as fold change vs controls. In bold the ones reaching the statistic significance.

**Supplementary Table 4** Primer sequences used for RT-qPCR

| Gene name | Forward | Reverse |
| --- | --- | --- |
| *Bcl2* | ATGACTGAGTACCTGAACCGGCAT | GGGCCATATAGTTCCACAAAGGCA |
| *Egr1* | GGGGAGCCGAGCGAACAAC | TGATGGGAGGCAACCGAGTC |
| *Ergic1* | ACGATGCCCTTTGACTTCAGG | ACGTAGAGCTCATTCACCACTT |
| *Fzd5* | CACAGGTACCTAGCTTGTCGT | TGATTAGGGCTCCGACTCCA |
| *Gapdh* | AGGTCGGTGTGAACGGATTTG | TGTAGACCATGTAGTTGAGGTCA |
| *Gnao1* | AATTACTCCTGCTGGGGGCT | CCATACTCCACGCCCAAAGT |
| *Hmga1* | CCAACTCCGAAGAGACCTCG | ATGCCCTCCTCTTCCTCCTT |
| *Ifit3* | CTGAACTGCTCAGCCCACA | TTCCCGGTTGACCTCACTCA |
| *Runx2* | TCGGAGAGGTACCAGATGGG | TGAAACTCTTGCCTCGTCCG |
| *Tg* | CATGGAATCTAATGCCAAGAACTG | TCCCTGTGAGCTTTTGGAATG |
| *Zfp36l2* | CCCTCGCCCGTTATTCATCT | CCAGGGATTTCTCCGTCTTG |
| *Zfp524* | CCCAGGCAGCATGCAAATG | GGCAGTAAGGCCAGAGGTTT |

Primers were designed using NCBI Primer Blast tool (http://www.ncbi.nlm.nih.gov/tools/primer-blast).
